# Navigating the 3D genome at single-cell resolution: techniques, computation, and mechanistic landscapes

**DOI:** 10.1093/bib/bbaf520

**Published:** 2025-10-06

**Authors:** Feitong Hong, Kaiyuan Han, Yuduo Hao, Wei Su, Xueqin Xie, Xiaolong Li, Qiuming Chen, Yijie Wei, Xinwei Luo, Sijia Xie, Benjamin Lebeau, Crystal Ling, Hao Lv, Li Liu, Hao Lin, Fuying Dao

**Affiliations:** The Clinical Hospital of Chengdu Brain Science Institute, School of Life Science and Technology, University of Electronic Science and Technology of China, Chengdu 610054, China; The Clinical Hospital of Chengdu Brain Science Institute, School of Life Science and Technology, University of Electronic Science and Technology of China, Chengdu 610054, China; The Clinical Hospital of Chengdu Brain Science Institute, School of Life Science and Technology, University of Electronic Science and Technology of China, Chengdu 610054, China; The Clinical Hospital of Chengdu Brain Science Institute, School of Life Science and Technology, University of Electronic Science and Technology of China, Chengdu 610054, China; The Clinical Hospital of Chengdu Brain Science Institute, School of Life Science and Technology, University of Electronic Science and Technology of China, Chengdu 610054, China; The Clinical Hospital of Chengdu Brain Science Institute, School of Life Science and Technology, University of Electronic Science and Technology of China, Chengdu 610054, China; The Clinical Hospital of Chengdu Brain Science Institute, School of Life Science and Technology, University of Electronic Science and Technology of China, Chengdu 610054, China; The Clinical Hospital of Chengdu Brain Science Institute, School of Life Science and Technology, University of Electronic Science and Technology of China, Chengdu 610054, China; The Clinical Hospital of Chengdu Brain Science Institute, School of Life Science and Technology, University of Electronic Science and Technology of China, Chengdu 610054, China; The Clinical Hospital of Chengdu Brain Science Institute, School of Life Science and Technology, University of Electronic Science and Technology of China, Chengdu 610054, China; School of Biological Sciences, Nanyang Technological University, Singapore 639798, Singapore; School of Biological Sciences, Nanyang Technological University, Singapore 639798, Singapore; The Clinical Hospital of Chengdu Brain Science Institute, School of Life Science and Technology, University of Electronic Science and Technology of China, Chengdu 610054, China; Yangtze Delta Region Institute (Quzhou), University of Electronic Science and Technology of China, Quzhou 324000, China; The Clinical Hospital of Chengdu Brain Science Institute, School of Life Science and Technology, University of Electronic Science and Technology of China, Chengdu 610054, China; School of Biological Sciences, Nanyang Technological University, Singapore 639798, Singapore

**Keywords:** single-cell 3D genomics, chromosome conformation capture techniques, computational frameworks, chromatin reprogramming

## Abstract

The 3D organization of the genome is critical for gene expression regulation, cellular identity, and disease progression. Traditional methods that analyze bulk genomic data often obscure cell-to-cell heterogeneity, limiting the resolution of intrinsic variability within complex biological systems. To overcome this, single-cell 3D genomics has emerged, revealing chromatin architecture at the individual cell level. Advanced experimental approaches enable genome-wide chromatin contact mapping, while computational frameworks reconstruct dynamic chromatin topologies from high-dimensional data. Building on these breakthroughs, recent advances in single-cell 3D genomics have led to transformative progress in epigenetics, linking 3D genome architecture with gene regulation, cellular identity, and disease phenotypes. This review focuses on the breakthroughs in single-cell 3D genomics, demonstrating how integrated experimental, computational, and mechanistic approaches decode chromatin architecture. These insights have deepened the understanding of genome function at the single-cell level and lay the foundation for future advances in precision medicine and topology-guided therapeutic strategies.

## Introduction

3D genome organization is essential for regulating gene expression, maintaining genome stability, and orchestrating cellular functions, such as DNA replication and repair [1–4]. Over the past decade, advances in chromosome conformation capture (3C) technologies—particularly Hi-C and its derivatives—have facilitated the mapping of genome-wide chromatin interactions in bulk cell populations [5, 6], revealing key organizational principles, including chromatin compartments [7], topologically associating domains (TADs) [8, 9], and regulatory loops [10]. However, these population-based methods obscure the cell-to-cell variability in chromatin architecture, a critical driver of cellular identity, plasticity, and disease phenotypes.

Single-cell 3D genomics has emerged as a transformative field that addresses the limitations of bulk analyses by resolving genome folding within individual nuclei [11]. This development has been driven by rapid progress in single-cell sequencing [12–16], high-resolution imaging, and microfluidic technologies [17–19]. These innovations allow for the investigation of chromatin structure in heterogeneous tissues, essential for understanding lineage decisions, disease mechanisms, and transcriptional regulation under various conditions.

A range of experimental platforms has been developed to profile chromatin conformation at single-cell resolution. Notably, approaches such as scHi-C, sci-Hi-C, and Micro-C have enabled genome-wide chromatin contact mapping in individual cells, revealing substantial variability in TAD boundaries, compartmentalization, loop dynamics, and underscoring the importance of structural heterogeneity in shaping gene regulatory programs [20–22]. Moreover, recent multi-omic protocols further integrate chromatin data with transcriptomic and epigenetic profiles, offering a holistic view of nuclear architecture and cellular function [23–27].

The high dimensionality, sparsity, and noise inherent in single-cell 3D genome data present major computational challenges. To address these, a variety of algorithms have been developed for quality control, normalization, contact imputation, structural reconstruction, and functional annotation. Notably, the rise of graph-based models, variational inference, and deep learning frameworks has enhanced the ability to infer 3D structures from sparse data, linking spatial genome architecture to gene regulation [28–34]. Based on these advancements, single-cell 3D genomics has redefined the ability to link spatial genome architecture with gene regulation in diverse biological contexts. From early embryonic development, cancer heterogeneity, and brain disorders to aging and stem cell fate decisions, single-cell 3D genomics has uncovered structural features that underpin phenotypic variation, transcriptional plasticity, and disease susceptibility [35–37]. These findings demonstrate that genome architecture is not merely a structural scaffold but an active regulatory layer that shapes cellular identity and function at multiple scales.

In this review, we provide a comprehensive overview of the current state of single-cell 3D genomics. We begin by summarizing key experimental innovations and technological platforms, followed by computational methodologies for data preprocessing, modeling, and structural reconstruction. Finally, we highlight recent mechanistic insights uncovered through single-cell 3D genomics across developmental, pathological, and evolutionary systems, and discuss future challenges and directions in this rapidly evolving field.

## Development of experimental techniques for single-cell 3D genomics

**Figure 1:**
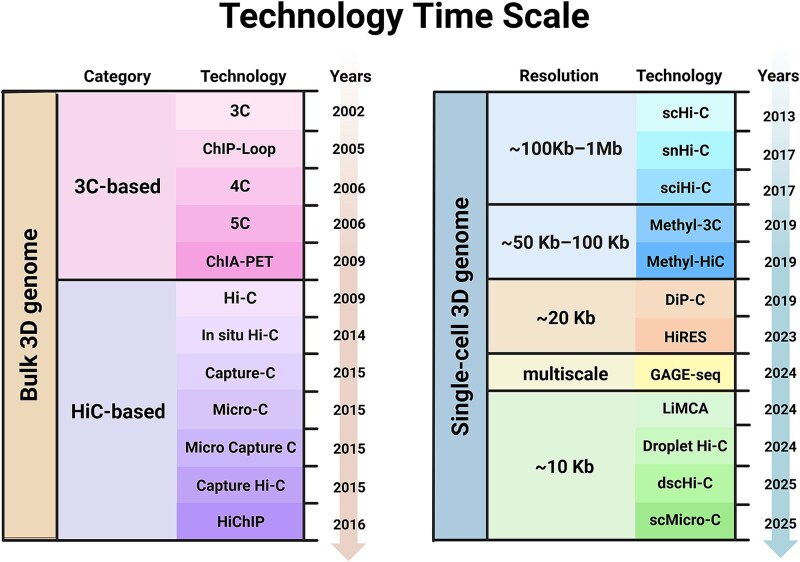
Timeline of 3D genome technology development (2002–2025), showing the progression from bulk to single-cell approaches and the advancement toward high-resolution methods.

3D genomics focuses on elucidating the spatial organization of chromatin within the cell nucleus and its implications for gene regulation, DNA replication, and repair [1, 38, 39]. Over the past decade, population-based bulk methods such as 3C, Hi-C, and their derivatives have provided comprehensive maps of chromatin interactions across diverse biological systems [7, 40–42]. These approaches have been widely adopted due to their reproducibility and applicability in a broad range of contexts, forming the foundation of genome architecture studies (Fig. 1 and Table 1).

**Table 1:** Summary of bulk 3D genome mapping technologies, providing an overview of representative methods with their year of development, experimental scope, achievable resolution, major strengths, and key limitations.

Technology	Year	Applicability	Resolution	Time/Cost	Advantages	Limitations
3C	2002	Locus‑specific	Locus‑level	Low cost, fast	Simple; ideal for validating specific point interactions	Cannot capture genome-wide interactions
4C	2006	Locus-to-genome	~10–100 kb	Moderate	Enables discovery of all interactions with a specific bait locus	Limited to one locus at a time
5C	2006	Multilocus within region	~1 Mb	Moderate–high	High-throughput within defined regions	Not genome-wide—requires complex design for each new region
ChIP-loop	2005	Protein-specific (local loops)	Locus-level	Moderate	Protein-mediated loop detection at selected loci	Limited resolution; antibody quality critical
ChIA-PET	2009	Protein-specific (genome-wide)	Pair-level	High cost, complex	Protein-centered, genome-wide interaction mapping	Expensive, heavily dependent on ChIP efficiency
HiC	2009	Genome-wide	~1–10 Mb	High depth, ~1 week of lab time	Unbiased genome-wide interaction map	High cost; complex data analysis
*In situ* HiC	2014	Genome-wide	~1 kb	High cost, optimized protocol	Improved specificity, can achieve kb-resolution	Still costly; data processing-intensive
Capture C	2015	Targeted genome-wide	sub-kb	Cost-effective for targets	High resolution in targeted regions	Limited to preselected targets; not comprehensive
Micro C	2015	Genome-wide	Nucleosome-level	Very high cost and data volume	Ultra-high resolution (nucleosome level)	Very expensive; data heavy
Capture Micro C	2015	Targeted genome-wide	Nucleosome-level	High cost	Ultra-high resolution in selected loci	Target-limited; not genome-wide
Capture HiC	2015	Targeted genome-wide	kb to sub-kb	Lower cost, efficient targeting	Cost-effective targeting of important regions (e.g. promoters)	Require probe design; less unbiased
HiChIP	2016	Targeted protein-wide	kb-level	Cost-effective, higher specificity	High resolution; protein-enriched, efficient loop detection	Dependent on antibody; richer interactions require more reads

Bulk data represent an average across millions of cells, which obscures heterogeneity and rare but functionally important chromatin configurations. The emergence of single-cell 3D genomics addresses this gap, enabling the resolution of chromatin folding at the individual-cell level and offering new opportunities to explore structural variability within heterogeneous tissues, dynamic developmental processes, and disease states (Fig. 1 and Table 2).

**Table 2:** Summary of single-cell 3D genome mapping technologies, providing an overview of major methods with their year of introduction, applicability, resolution, sequencing cost, sequencing depth, advantages, and limitations.

Technology	Year	Applicability	Resolution	Time/Cost	Depth	Advantages	Limitations
scHi-C	2013	Genome-wide	~100 kb–1 Mb	Moderate cost; low throughput	~10^4^–2 × 10^5^	Simple protocol, preserves single-cell structure	Sparse data, low throughput
snHi-C	2017	Genome-wide	~100 kb–1 Mb	Moderate cost; low throughput	~1.9 × 10^6^	Simple protocol, preserves single-cell structure	Sparse data, low throughput
sci-Hi-C	2017	Genome-wide	~100 kb–1 Mb	Moderate cost, Moderate time	~10^3^	High throughput via combinatorial indexing	Barcode complexity, still sparse
Methyl-3C	2019	Genome-wide	~50 kb–100 kb	Moderate cost	~10^2^	Integrates DNA methylation with spatial contacts	Complex library prep; bisulfite harshness
Methyl-HiC	2019	Genome-wide	~50 kb–100 kb	Moderate cost	~10^2^	Integrates DNA methylation with spatial contacts	Complex library prep; bisulfite harshness
DiP-C	2019	Genome-wide (allele-specific)	~20 kb	Time consuming; low throughput	~10^5^	Very high resolution (~20 kb), allele-specific	Low throughput, computationally intensive
HiRES	2023	Genome-wide + scRNA	~20 kb	Excess cost and complexity	~3 × 10^5^	Structural + transcriptome data per cell	Complex, multi-omics integration
GAGE-seq	2024	Genome-wide + multi-modal	multiscale	Moderate high cost	~10^3^–10^4^	Captures structure and expression in intact cells	Deep sequencing needed, index care required
LiMCA	2024	Genome-wide + full RNA	~10 kb	High cost	~10^2^	High contact counts + full-length transcripts	Labor-intensive (nuclear/cytoplasmic split)
Droplet Hi-C	2024	Genome-wide	~10 kb	Low cost per cell; high throughput	~10^4^	Fast (~10 h), scalable for large sample sets	Microfluidics required
dscHi-C	2025	Genome-wide	~10 kb	High throughput; high sensitivity	~3 × 10^4^	Ultra-high throughput and sensitivity	Requires droplet microfluidics
scMicro-C	2025	Genome-wide	~10 kb	High cost; low throughput	~10^3^–10^4^	Reveals PESs and multi‑enhancer hubs	High sequencing depth; experimental complexity; low throughput

### Single-cell 3D genomics derived from bulk technologies

Bulk 3D genome mapping assays use a common chemical framework of formaldehyde crosslinking, enzymatic fragmentation, proximity ligation, and sequencing, but require large inputs that hinder the detection of cell-specific features. To enable single-cell resolution, researchers have adapted these bulk protocols by introducing innovations such as miniaturized reaction volumes, refined nuclear isolation, molecular barcoding, and microfluidics. These adaptations preserve the core chemistry of their bulk predecessors while making it possible to profile chromatin contacts in thousands of individual cells with high resolution and throughput.

The following section presents a chronological overview of major single-cell 3D genomics methods derived from bulk frameworks, highlighting their methodological origins, technical advances, and performance characteristics.

#### From bulk to single-cell: methodological evolution of scHi-C, snHi-C, sci-Hi-C, and scMicro-C

Bulk 3D genome technologies provides an averaged view of chromatin interaction profiles across large populations of cells, thereby obscuring the intrinsic cell-to-cell variability that is essential for understanding dynamic chromatin structures. To overcome this limitation, a range of single-cell adaptations—such as single-cell Hi-C (scHi-C), single-nucleus Hi-C (snHi-C), single-cell combinatorial indexed Hi-C (sci-Hi-C), and more recently single-cell Micro-C (scMicro-C)—have been developed, each preserving the fundamental chemistry of bulk assays while extending their capabilities to resolve genome architecture at the individual-cell level.

scHi-C emerged to address cellular heterogeneity by profiling chromatin architecture at the single-cell level [20]. By combining glass capillary-based nuclear isolation with adapted *in situ* Hi-C protocols, scHi-C minimizes cross-nuclear contamination and generates high-resolution libraries with interaction counts ranging from several tens of thousands to over 200 000 per cell, depending on sequencing depth and cell quality [22, 43, 44]. Such resolution makes it possible to study cell-type-specific folding patterns, dynamic reorganization during differentiation, and rare configurations that may be masked in population-averaged data. Nonetheless, scHi-C data are intrinsically sparse, with most cell–locus pairs unobserved, necessitating specialized computational approaches for noise reduction and bias correction.

Expanding on the advancements of scHi-C, snHi-C was developed to utilize isolated nuclei rather than intact cells, thereby streamlining sample preparation and extending the applicability of the technique to challenging specimens, including frozen tissues, oocytes, and zygotes [45]. snHi-C preserves the integrity of chromatin structure while maintaining single-cell resolution, enabling the study of chromatin features like loops, TADs, and compartments at the individual cell level. Notably, both scHi-C and snHi-C overcome the averaging bias of bulk-cell assays, making them indispensable tools for investigating cellular heterogeneity [35], tumorigenesis [46], and other processes driven by cellular diversity [47, 48]. While snHi-C broadens the applicability of scHi-C to otherwise inaccessible samples, it shares the limitations of scHi-C, including high technical variability and limited per-cell coverage.

sci-Hi-C addresses the throughput constraints of scHi-C by replacing physical isolation with split–pool combinatorial barcoding, enabling parallel processing of thousands of nuclei in a single experiment [22]. Each round of barcoding exponentially increases the number of uniquely indexable cells, allowing cost-effective profiling of large populations while maintaining the *in situ* ligation chemistry. This scale facilitates robust statistical analysis of cell-to-cell variability in chromatin folding, including rare subpopulation-specific features. However, the increased multiplexing can introduce batch effects and demands stringent barcode design and error correction to avoid cross-cell misassignment.

Beyond Hi-C derivatives, advances in micrococcal nuclease (MNase)-based approaches have further expanded single-cell 3D genomics. The high-resolution advantages of Micro-C in bulk experiments—achieved by substituting restriction enzymes with MNase digestion to reach nucleosome-level mapping—have inspired its adaptation to single-cell analysis [21]. Micro-C provides genome-wide chromatin interaction maps at nucleosome resolution, Capture-C enriches for specific loci using hybridization probes to increase effective depth, and Capture Micro-C combines these strategies to achieve targeted, nucleosome-level interrogation of chromatin architecture [49–51]. Building on these principles, scMicro-C integrates MNase-based fragmentation with low-input workflows optimized for single cells, achieving an improved spatial resolution of ~5-kb [52]. This advance enables the identification of specialized 3D regulatory architectures, such as promoter–enhancer stripes (PESs), which connect a gene’s promoter to multiple enhancers through cohesin-mediated loop extrusion. At single-cell resolution, scMicro-C reveals the prevalence of multi-enhancer hubs, where multiple enhancers spatially cluster with their target promoter, offering mechanistic insight into how enhancers are coordinated to regulate gene expression in specific cellular contexts. While scMicro-C retains the fine-scale structural resolution of bulk Micro-C, it uniquely captures cell-to-cell variability in enhancer–promoter communication, although at the cost of high sequencing depth requirements and considerable experimental complexity.

Collectively, scHi-C, snHi-C, sci-Hi-C, and scMicro-C illustrate the diverse strategies employed to adapt bulk Hi-C and Micro-C chemistries to the single-cell context. Each platform addresses specific experimental challenges—whether resolution, sample accessibility, fine-scale structural detection, or throughput—expanding the scope of 3D genomics to encompass heterogeneous and rare cell populations. These methodological advances have not only deepened understanding of structural variability in the genome but also set the stage for multi-omic and functional integration at the single-cell level.

#### Droplet-based single-cell Hi-C platforms: droplet Hi-C and dscHi-C

While plate-based and combinatorial indexing scHi-C methods have substantially advanced the resolution and throughput of chromatin conformation profiling, their labor-intensive workflows and limited scalability remain barriers to large-scale studies. To overcome these constraints, droplet-based adaptations integrate the core chemistry of bulk Hi-C with high-throughput microfluidic partitioning. By encapsulating individual nuclei with processing reagents in picoliter droplets and introducing barcoding strategies, these methods enable massively parallel single-cell 3D genome mapping. This section outlines the working principles, performance characteristics, and comparative strengths of Droplet Hi-C and dscHi-C, emphasizing their roles in expanding single-cell 3D genomics to large and heterogeneous cell populations.

Droplet Hi-C addresses the throughput and scalability limitations of earlier scHi-C formats by combining *in situ* 3C with droplet microfluidics [53]. In this system, individual nuclei are encapsulated in picoliter droplets together with reagents for fragmentation, proximity ligation, and barcode tagging. Hydrodynamic focusing is used for precise nuclear alignment, while viscoelastic fluid-controlled droplet generation ensures consistent single-nucleus capture. Barcode-linked transposase complexes uniquely index chromatin fragments, achieving ~98.7% encapsulation efficiency and <5% doublet rate. By performing chromatin processing entirely within droplets, the method minimizes cross-contamination and preserves native chromatin structure. Capable of profiling over 10 000 cells per experiment at resolutions down to 50-kb, Droplet Hi-C has been applied to complex tissues such as the mouse brain and to cancer models for detecting abnormal genome organization and extrachromosomal DNA (ecDNA) landscapes, as well as studying mitotic chromosome condensation and its relationship to cell cycle phases.

Droplet single-cell Hi-C (dscHi-C) builds on the droplet framework by incorporating combinatorial indexing, increasing scalability to over 50 000 cells per experiment while maintaining high resolution [54]. Using a dual-barcode system—spatial and temporal identifiers—linked to Tn5 transposase, dscHi-C enables unambiguous cell assignment and improved dynamic tracking. Applied to the developing mouse brain, it has profiled over 30 000 single cells across developmental stages, uncovering age-dependent chromatin reorganization, with affected genes enriched in neuron-specific metabolic and morphological pathways and innate immune responses in glial cells. Its multi-omics extension, dscHi-C-multiome, simultaneously captures chromatin architecture and gene expression, enabling direct mapping between structural genome features and transcriptional programs.

When considered together, Droplet Hi-C and dscHi-C represent complementary adaptations of droplet-based single-cell 3D genomics. Droplet Hi-C is optimized for capturing detailed chromatin folding and cell cycle-associated structural changes, making it well suited for rare or dynamic cell states. In contrast, dscHi-C emphasizes large-scale profiling and integration with transcriptomics, which supports mapping developmental trajectories and resolving tissue heterogeneity. Both approaches expand the experimental toolkit for studying chromatin architecture at single-cell resolution, though they require substantial sequencing depth and robust bioinformatic pipelines to address the inherent sparsity and noise of droplet-based Hi-C datasets.

### Evolution of single-cell 3D genomics: from single-omics to multimodal integration

The emergence of single-cell multi-omics technologies has ushered in a transformative era for exploring the dynamic interplay between 3D genome architecture and transcriptional regulation. Recent methodological advancements have enhanced not only the resolution of chromatin interactions but also the integration of epigenetic, transcriptional, and spatial genomic information within individual cells. These innovations are reshaping the understanding of how nuclear topology orchestrates cell-type-specific gene expression programs and contributes to phenotypic diversity across biological systems.

#### Structural genomics and spatial reconstruction

For multi-omic integration to yield biologically meaningful insights, it benefits from being anchored in an accurate and high-resolution representation of the underlying 3D genome architecture. Structural genomics approaches address this need by reconstructing nuclear organization at kilobase-scale resolution, resolving allele-specific configurations, and capturing structural heterogeneity between cells. These detailed spatial frameworks provide an important foundation for contextualizing epigenomic, transcriptomic, and other molecular layers, thereby enabling a more precise interpretation of genome function.

Dye-labeling *in situ* Proximity Chromatin Capture (DiP-C) exemplifies structural genomics approaches by combining *in situ* chromatin conformation capture with diploid genome assembly to generate allele-resolved 3D genome maps at ~20-kb resolution [55, 56]. The workflow begins with fluorescence-guided chromatin proximity labeling, which selectively tags spatially adjacent DNA segments to enhance the specificity of interaction detection and reduce background ligation events. Following proximity ligation, optimized whole-genome amplification minimizes polymerase-induced bias while preserving chromatin topology, enabling reliable estimation of contact frequencies. The sequencing output is subsequently processed using computational phasing algorithms to separate homologous chromosomes, allowing visualization of allele-specific nuclear territories within single cells.

This capability to correlate dynamic structural changes with allele-specific transcriptional activity makes DiP-C particularly suited for investigating processes such as developmental allele switching, regulatory asymmetry, and disease-associated structural variation [57]. Beyond high-resolution structural reconstruction, the method offers opportunities for integration with additional molecular readouts, including transcriptomic and epigenomic profiles, to explore how allele-specific genome features influence functional regulation. Nevertheless, achieving sufficient per-cell coverage requires substantial sequencing depth, and the reliance on fluorescence-based labeling and computationally intensive phasing can limit scalability. Compared with other single-cell 3D genomics platforms, DiP-C provides superior allele resolution and structural fidelity, albeit with trade-offs in throughput and experimental accessibility.

#### Epigenomics integration

Building on the structural frameworks established by high-resolution 3D genome mapping, a growing set of single-cell methods now integrate chromatin conformation assays with epigenomic readouts to investigate how chemical modifications of DNA and histones, as well as chromatin accessibility, influence nuclear architecture. These approaches facilitate the direct correlation of spatial genome organization with regulatory states, offering an integrated perspective on the bidirectional relationship between epigenetic landscapes and 3D chromatin topology. Such integrative measurements are particularly valuable for dissecting cell-type-specific regulatory mechanisms and for uncovering how epigenetic variation contributes to cellular heterogeneity.

The integration of chromatin conformation capture with DNA methylation profiling was realized in 2019 through the development of Methyl-3C and Methyl-HiC, which enable the examination of the spatial and epigenetic interface at single-cell resolution. Methyl-3C combines 3C-based ligation with bisulfite sequencing to detect both chromatin contacts and the methylation status of interacting loci [24]. This targeted strategy enables the reconstruction of methylation-anchored interaction networks, revealing how locus-specific methylation patterns influence 3D chromatin folding during processes, such as cellular differentiation. Methyl-HiC extends this principle to a genome-wide scale, producing base-resolution methylomes alongside high-coverage chromatin contact maps from the same nucleus [25]. By correlating methylation states with features such as A/B compartmentalization and loop formation, Methyl-HiC has provided evidence for methylation-dependent chromatin compartment switching in specific cell subtypes. Together, these methods provide complementary scales of analysis—Methyl-3C for high-confidence, locus-focused interrogation, and Methyl-HiC for comprehensive methylome–structure integration—while requiring careful optimization of sequencing depth to balance coverage and resolution.

To address the lack of transcriptional correlation in conventional scHi-C assays, Liu *et al*. [43] developed Hi-C and RNA-seq Employed Simultaneously (HiRES), a sequencing-based platform that profiles single-cell transcriptomes and 3D genome architectures in parallel. HiRES incorporates *in situ* reverse transcription within a 3C workflow, followed by flow sorting of intact single cells and parallel amplification of genomic DNA and RNA. This design enables barcode-matched pairing of chromatin contact maps and transcriptome profiles from the same cell, as well as allele-specific interaction network reconstruction at 50-kb resolution. By retaining reverse-transcribed cDNA within the nucleus before lysis, HiRES can also capture nascent RNA transcripts, providing a snapshot of active transcription in the context of local chromatin structure. Applied to thousands of single cells from developing mouse embryos, HiRES revealed coordinated changes in chromatin compartmentalization and transcriptional programs across developmental stages [58]. The method is adaptable to diverse cell types and can be integrated with other epigenomic measurements, making it a valuable approach for examining the interplay between spatial genome organization and transcriptional regulation at single-cell resolution.

#### Transcriptomics integration

While epigenomic integration has provided important insights into how chromatin states correlate with genome topology, understanding the functional outcomes of these structural variations requires parallel measurement of transcriptional activity. Single-cell approaches that simultaneously capture 3D chromatin interactions and transcriptome profiles allow researchers to link spatial genome features directly to gene expression programs within the same cell. This dual-modal perspective is particularly valuable for resolving transcriptional heterogeneity, identifying structure–function relationships, and tracking dynamic regulatory changes during development, differentiation, or disease progression.

Linking mRNA to Chromatin Architecture (LiMCA) was developed by Wu *et al*. [59] to address limitations observed in HiRES, including reduced sensitivity from genomic DNA damage during reverse transcription, contamination from cytoplasmic RNA, and incomplete transcript coverage limited to 3′ ends. LiMCA physically separates the nucleus and cytoplasm of the same single cell prior to processing, allowing independent assessment of chromatin structure and transcriptome while preserving the integrity and detection performance of both modalities. In the nuclear fraction, chromatin conformation capture is performed under conditions optimized to maintain 3D topology, while the cytoplasmic fraction is processed for full-length mRNA sequencing. This design eliminates cross-compartment interference, increases gene detection rates by ~30%, and yields ~1 million valid Hi-C contacts per cell. LiMCA has been applied to characterize enhancer–promoter rewiring during cellular reprogramming, allele-specific looping in aneuploid cancer cells, and spatial coordination of olfactory receptor gene clusters. However, by physically separating nuclear and cytoplasmic compartments, LiMCA loses the ability to measure the spatial coordination between cytoplasmic mRNA localization and nuclear architecture, which may be relevant in certain regulatory contexts.

Genome Architecture and Gene Expression sequencing (GAGE-seq) addresses a different set of challenges by maintaining the physical coupling between nuclear chromatin structure and gene expression states [60]. Instead of separating nuclear and cytoplasmic contents, GAGE-seq combines combinatorial indexing, *in situ* reverse transcription, and chromatin conformation capture within intact cells. This approach enables simultaneous generation of chromatin contact maps and transcriptomes without disrupting spatial associations, facilitating parallel profiling of thousands of single cells. In mouse cortex and human bone marrow CD34^+^ cells, GAGE-seq has revealed that multiscale 3D genome features—ranging from compartment shifts to fine-scale looping—inform cell-type-specific transcriptional programs and can be linked to regulatory elements via co-detected expression profiles [60]. Integration with spatial transcriptomics has further uncovered region-specific variation in genome architecture in the mouse brain, while studies of hematopoietic differentiation have shown asynchronous changes between chromatin topology and transcriptional output, suggesting that genome reorganization and transcriptional reprogramming can be temporally decoupled. Despite its scalability and cost-effectiveness, GAGE-seq requires high sequencing depth to ensure adequate per-cell coverage, and the reliance on combinatorial indexing can introduce barcode collisions if library complexity is not carefully managed.

## Computational approaches for single-cell 3D genomic data analysis

Following the experimental methodologies outlined in the previous section, which generate single-cell 3D genomic data through diverse chemical, molecular, and sequencing innovations, the next crucial step is to translate these raw outputs into meaningful biological insights. This transition from wet-lab generation to dry-lab interpretation requires computational approaches specifically designed to address the unique challenges of single-cell 3D genomics, including extreme sparsity, high dimensionality, and pronounced cell-to-cell variability.

**Figure 2:**
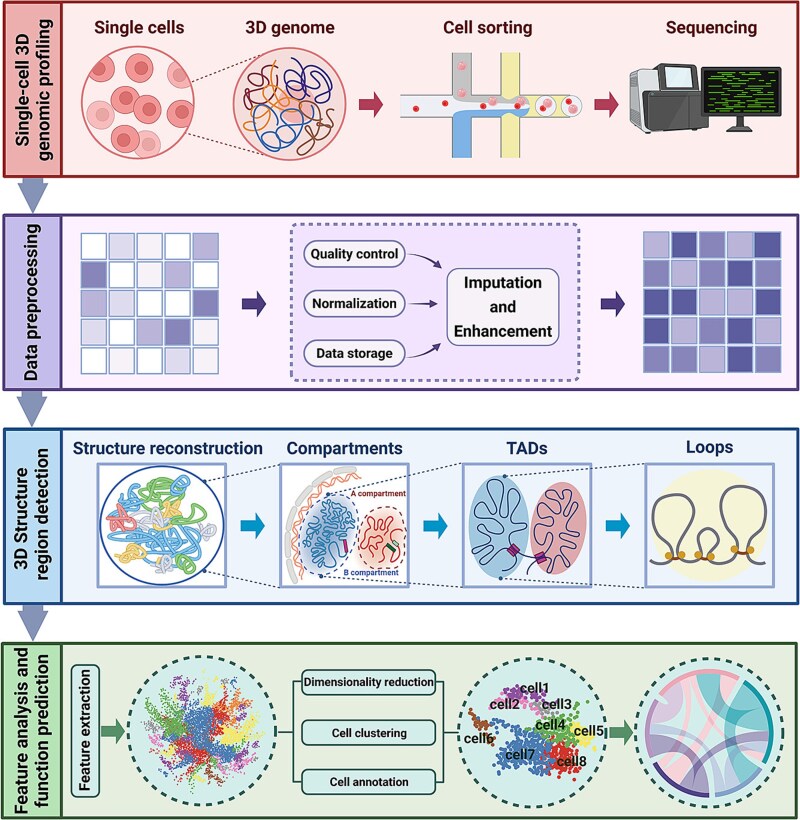
Framework for single-cell 3D genome analysis, capturing key stages from profiling and preprocessing to feature extraction, classification, and functional prediction.

Before delving into the details of individual methods, it is important to define the overarching goals and applicability of these computational strategies. Broadly, they aim to ensure data quality, recover missing information, reconstruct spatial chromatin structures, detect functional genomic domains, extract informative features, and integrate complementary omics layers for functional prediction. Figure 2 illustrates the end-to-end analytical workflow, from single-cell 3D genome profiling and data preprocessing to 3D structure and region detection and feature analysis and functional prediction. In parallel, Table 3 summarizes each major computational process, outlining its primary objectives, representative tools, and typical use cases. Together, these resources bridge experimental generation and computational interpretation, offering readers both a conceptual overview and a practical guide for navigating the rapidly advancing landscape of computational single-cell 3D genomics.

**Table 3:** Summary of computational approaches for single-cell 3D genomic data analysis, providing an overview of strategies for preprocessing, imputation, structure reconstruction, clustering, and functional annotation.

Process	Goal	Representative tools	Applicability
Data preprocessing	Ensure analytical accuracy by removing noise, correcting biases, and optimizing storage	Quality control: GiniQC and hicQuickQC;	Essential first step for all scHi-C datasets; ensures data comparability, efficiency, and integrity
		Normalization: scHiCNorm, BandNorm, and scVI-3D;	
		Data storage: Scool (Cooler extension)	
Data imputation and enhancement	Recover missing interactions and improve resolution of sparse scHi-C data	Traditional methods: scHiCluster and HiCImpute;	Increases data usability for downstream analysis; improves detection of loops, compartments, and structural features
		Deep learning: Higashi, HiC-SGL, scDEC-Hi-C, ScHiCEDRN, ScHiCAtt, scCTG, HiCENT, and ImputeHiFI	
3D structure reconstruction	Rebuild 3D chromatin conformation from contact maps	Physics-based models: RPR, LJ3D, DPDchrom, MBO, SCL, and MaxEnt;	Enables visualization and mechanistic study of chromatin folding and nuclear organization
		Statistical models: ISD, Si-C, and SIMBA3D;	
		Deep learning: HiC-GNN, ChromoGen, and SnapHiC-G	
Domain and functional region detection	Identify genomic compartments, TADs, and loops	A/B compartments: Spectral Method with Non-Backtracking and scGHOST;	Links structural organization to gene regulation; facilitates comparative analysis across cell types and conditions
		TADs: deTOKI, deDoc2, HiCS, scKTLD, and DiffDomain;	
		Loops: SnapHiC, SnapHiC2, SnapHiC-D, and scHiCDiff	
Feature extraction and dimensionality reduction	Derive low-dimensional, informative features from sparse contact maps	Topic modeling: LDA (scHiC topic modeling);	Improves clustering, classification, and visualization of structural variability
		Graph embedding: scHiCEmbed, scGAD, and scENCORE	
Cell annotation and clustering	Classify cells based on chromatin architecture	Traditional clustering: scHiCExplorer;	Supports identification of cell types and states, revealing structure–function relationships
		Deep learning: scHiCluster, scHiCStackL, SCANN, CTPredictor, scHiClassifier, and Fast-Higashi	
Multi-omics integration and functional prediction	Integrate 3D genome data with other omics for functional insights	Multi-omics: MUDI and Muscle;	Enables linking chromatin structure to transcriptional activity, DNA methylation, and other cellular phenotypes
		Functional prediction: scHiMe and scHiGex	

### Data preprocessing

Computational analysis of single-cell 3D genomic data aims to extract biologically meaningful information from inherently sparse and noisy chromatin contact maps. Before delving into individual analytical steps, it is essential to clarify that the overall goal is to transform raw sequencing outputs into reliable, interpretable representations of chromatin organization, enabling downstream tasks such as 3D structure reconstruction, domain detection, cell classification, and functional inference. This process typically begins with data preprocessing, which addresses technical noise, corrects systematic biases, and optimizes data storage to ensure analytical accuracy and reproducibility. In the context of scHi-C, preprocessing is particularly critical due to the high variability across individual cells. The following subsections outline key preprocessing steps, including quality control, normalization, and efficient storage strategies (Table 3).

#### Quality control

As the foundational step in scHi-C data processing, rigorous quality assessment serves as a critical checkpoint, ensuring data integrity and biological relevance while addressing challenges posed by data sparsity, technical variability, and inherent noise in single-cell chromatin interaction profiles. Recognizing the limitations of traditional metrics focused solely on read counts, the GiniQC framework offers a more comprehensive evaluation by combining the percentage of valid cis read pairs with the Gini index, which quantifies the uneven distribution of chromatin contacts across the genome [61]. Higher GiniQC values generally indicate superior data quality, reflecting balanced genomic coverage and minimal technical artifacts. For scalable assessments, hicQuickQC provides a computationally efficient alternative by estimating quality metrics from a subset of reads [62]. While hicQuickQC sacrifices some resolution for speed, making it ideal for preliminary screening or iterative experimental optimization. Benchmarking studies show that GiniQC is more sensitive in detecting subtle quality degradations, whereas hicQuickQC excels when computational resources are limited.

#### Normalization

Normalization corrects biases from genomic distance, sequencing depth, and cell-specific noise. However, traditional bulk Hi-C normalization methods fail to account for the zero-inflation in scHi-C data. To this end, Liu and Wang [63] developed scHiCNorm, which utilizes zero-inflated and hurdle models to correct biases from scHi-C data. Additionally, methods like BandNorm and scVI-3D provide more robust approaches by modeling zero-inflation, sparsity, and batch effects, improving data reliability for tasks such as clustering and lineage inference [64]. While scHiCNorm is robust for bias correction in general datasets, it may be less effective for highly sparse data compared with scVI-3D. BandNorm is advantageous in large-scale datasets where computational efficiency is critical, but its performance may plateau in extremely sparse conditions. Benchmarking across synthetic and experimental datasets suggests that scVI-3D consistently achieves the highest clustering accuracy under heterogeneous cell populations, whereas BandNorm dominates in runtime efficiency.

#### Data storage

The high dimensionality and sparsity of scHi-C data present significant storage challenges. To address this issue, Wolff *et al*. [65] introduced the *Scool* format—an extension of the Cooler framework—designed to store multiple sparse matrices within a single HDF5 file. This hierarchical structure reduces storage requirements by over 50% compared with dense formats, improving data accessibility and supporting high-throughput, reproducible workflows. Compared with dense storage, *Scool* offers vastly superior scalability and interoperability with mainstream tools. However, for workflows requiring frequent random access to individual contact matrices at multiple resolutions, dense formats may still offer marginally faster retrieval times at the cost of large storage overhead. Benchmarking in multiterabyte datasets has shown that *Scool* maintains I/O performance with minimal degradation, making it preferable for collaborative and reproducible analyses.

### Data imputation and enhancement

A key analytical challenge in single-cell 3D genomics is that raw scHi-C contact maps are extremely sparse, with the vast majority of possible chromatin interactions unobserved due to limited sequencing depth. This sparsity not only obscures fine-scale structural features such as promoter–enhancer loops and TAD boundaries, but also reduces the statistical power of downstream analyses, including 3D structure reconstruction, domain detection, and cell-type classification. The goal of imputation and enhancement is, therefore, to infer the missing contacts and sharpen the spatial resolution of scHi-C data, enabling a more accurate and comprehensive view of chromatin architecture. The inherent sparsity and technical noise in scHi-C data necessitate advanced imputation and enhancement strategies to recover missing interactions and improve spatial resolution. This section provides an overview of traditional, deep learning-based, and emerging approaches for imputation and resolution enhancement in scHi-C data (Table 3).

#### Traditional imputation

Traditional imputation approaches for scHi-C data primarily rely on mathematical frameworks. One such method is scHiCluster, which employs linear convolution and random walk with restart (RWR) to impute contact matrices, followed by principal component analysis (PCA) [66]. While effective for clustering low-coverage datasets, it has limited ability to capture cell-type-specific interactions. In contrast, HiCImpute utilizes a Bayesian hierarchical model to integrate single-cell and bulk Hi-C data, improving imputation in sparse regions [67]. Benchmarking studies suggest that while both methods improve data usability, scHiCluster is more appropriate for large datasets where computational efficiency is a priority, whereas HiCImpute is better suited for targeted analyses that tolerate higher computational costs but demand more accurate recovery of sparse interactions. However, traditional methods often struggle to capture the nonlinear complexity of chromatin interactions, motivating deep learning methods.

#### Deep learning-based methods

Recent advances in deep learning have revolutionized scHi-C data imputation by capturing high-dimensional and long-range chromatin interaction patterns. One representative example is Higashi, which introduces hypergraph-based representation learning by modeling both cells and genomic loci as nodes to enhance local specificity and global coherence in imputation [68]. This enables improved reconstruction of both short- and long-range interactions, though its memory and computational demands may be prohibitive for extremely large datasets. Building on this concept, HiC-SGL introduced a three-stage graph neural networks (GNNs) architecture, improving completion accuracy by 22% and generating informative low-dimensional embeddings for the analysis of structural variation across cell types [69]. Its performance is most notable in moderately to highly covered datasets, but can be less stable in ultra-sparse scenarios where GNN embeddings lack sufficient signal. The advent of scDEC-Hi-C marked a methodological shift toward end-to-end generative modeling to address higher-dimensional data complexity [70]. scDEC-Hi-C leverages a variational autoencoder to disentangle cell-type-specific chromatin features using hierarchical graph attention. This method excels at modeling heterogeneity but requires careful hyperparameter tuning and large training sets to avoid overfitting. Similarly, GAN-based approaches like ScHiCEDRN combines residual neural networks with adversarial learning to simulate multiscale chromatin architecture with strong generalization across cells and cell types [71].

In recent years, the integration of attention mechanisms has propelled a new wave of innovation in scHi-C analysis, improving dependency modeling and multimodal integration [72]. ScHiCAtt pioneers this direction by introducing multihead self-attention to enhance long-range genomic interaction, outperforming conventional imputation methods by 29%, particularly in resolving elusive promoter–enhancer interactions [73]. Another notable contribution is scHi-C to geometry (scC_T_G), which involves convolving the raw scHi-C matrix prior to applying the C_T_G algorithm [74, 75]. This approach effectively reduces the sparsity of the matrix, facilitating 3D chromatin structure reconstruction. Advancing this progression, the transformer-based HiCENT integrates CNNs for local feature extraction and transformers for modeling complex spatial interactions, enhancing contact map resolution and clustering [76]. Complementing these methods, another notable advancement is the integration of multimodal data, exemplified by ImputeHiFI [77]. This approach combines scHi-C with RNA fluorescence *in situ* hybridization for high-fidelity spatial imputation of chromatin loci, surpassing the limitations of unimodal approaches.

In summary, while deep learning methods significantly expand the capacity to model sparse and heterogeneous data, their optimal performance depends on dataset size, coverage depth, and computational resources. Benchmarking studies indicate that GNN-based methods like HiC-SGL offer balanced scalability and accuracy, generative models (e.g. scDEC-Hi-C and ScHiCEDRN) excel in capturing nonlinear patterns, and multimodal approaches like ImputeHiFI achieve the highest robustness when auxiliary data are available. To ensure reproducibility and fair comparison, standardized evaluation protocols—including controlled sparsity levels, uniform training/testing splits, and shared reference datasets—are critical, particularly for emerging deep learning frameworks.

### 3D structure reconstruction

The refinement of sparse and noisy contact maps through imputation and resolution enhancement lays the foundation for an equally critical analytical stage—reconstructing the spatial organization of chromatin. In the single-cell context, this task seeks to transform processed interaction matrices into accurate three-dimensional models that capture both global genome architecture and cell-specific structural variability. Such reconstructions are essential for elucidating how chromatin folding influences gene regulation, nuclear compartmentalization, and dynamic biological processes including cell differentiation and stress responses. This section reviews recent 3D genome reconstruction methods, which can be broadly grouped into physics-based simulations, Bayesian frameworks, and deep learning-based approaches (Table 3).

#### Physics-based models

Physics-based simulations leverage biophysical principles to model chromatin folding dynamics. Early work by Hirata *et al*. [78] introduced a nonlinear time-series analysis tool named RPR for reconstructing 3D chromosome structures from Hi-C contact maps, offering a relatively simple entry point but limited in capturing high-resolution details. To improve physical realism, Zha *et al*. [79] proposed LJ3D, which employs a Lennard–Jones potential and 2D Gaussian-based contact probabilities, and uses Metropolis–Hastings and simulated annealing to generate physically stable structures, though at significant computational cost for large genomes. Another notable technology is DPDchrom, which employs dissipative particle dynamics to simulate spring-like relaxation between contact sites, effectively capturing the polymeric behavior of chromatin in 3D space, but requires careful parameter tuning to avoid nonbiological conformations [80].

Other methods like manifold based optimization (MBO) modeled chromatin as an elastic polymer chain constrained on Riemannian manifolds, achieving a 73% speed improvement over traditional approaches such as LJ3D [81]. Single-cell lattice (SCL) introduced a voxel-based discrete framework with energy-guided Monte Carlo sampling, delivering high structural accuracy and a five-fold increase in computational efficiency [82]. MaxEnt further advanced the field by reconstructing conformational ensembles through entropy-maximized spatial distributions, incorporating Bayesian uncertainty and multiresolution modeling [83].

Overall, physics-based methods are best suited for studies prioritizing physical plausibility and mechanistic modeling, especially when contact data are relatively dense. However, they can be computationally demanding and less robust in ultra-sparse single-cell datasets.

#### Statistical methods

Statistical approaches estimate distributions to capture chromatin heterogeneity and uncertainty, allowing explicit modeling of uncertainty and heterogeneity across cells. For example, inference of structural determination (ISD) employs Bayesian inference with Markov Chain Monte Carlo (MCMC) sampling to estimate conformational distributions [84]. It also enables the mapping of epigenetic features in 3D space. Si-C extends Bayesian modeling for super-resolution reconstruction across entire genomes [85]. It combines ISD and NucDynamics principles, effectively generating biologically and statistically consistent chromatin structures. Additionally, SIMBA3D is a Bayesian framework that uses multiscale optimization and Broyden–Fletcher–Goldfarb–Shanno (BFGS) routines to capture hierarchical variations in chromatin architecture by balancing global topological consistency with local contact fidelity [86].

Compared to physics-based simulations, statistical methods offer greater flexibility in handling sparse and noisy data, and their probabilistic outputs are advantageous for hypothesis testing. However, they may underperform in reproducing physically realistic dynamics without additional biophysical constraints.

#### Deep learning-based methods

Deep learning frameworks aim to overcome the limitations of both physics-based and statistical methods by learning complex, nonlinear mappings from contact maps to spatial coordinates. For example, HiC-GNN leverages graph convolutional networks to infer population-level 3D chromatin structures, achieving efficiency and generalizability beyond conventional methods [87]. However, predicting single-cell 3D conformations remains challenging due to the pronounced structural heterogeneity among individual cells. Another method like ChromoGen uses a generative framework combining transformer encoders and diffusion-based decoders to generate statistically diverse and biologically plausible conformations, bypassing the inefficiencies of polymer simulations [88]. To further improve resolution and regulatory inference, SnapHiC-G integrates GANs, GNNs, and global background modeling to detect long-range enhancer–promoter interactions based on scHi-C data, outperforming existing methods in resolving cell-type-specific chromatin loops [89].

Deep learning approaches generally excel in resolution enhancement and cross-cell-type generalization, and they can integrate seamlessly with imputation methods from “Data imputation and enhancement” Section. Nonetheless, their black-box nature, dependence on large datasets, and variable reproducibility across runs highlight the need for standardized benchmarks.

### Domain and functional region detection

Following the reconstruction of 3D chromatin structures, an equally important analytical stage is the identification of functional genomic domains—such as A/B compartments, TADs, and chromatin loops—that mediate the interplay between genome architecture and transcriptional regulation. The overarching goal of this step is to delineate structural units of the genome, quantify their variability across cells or conditions, and link these patterns to functional outcomes such as gene activation or repression. In scHi-C data, this task is complicated by extreme sparsity and technical noise, requiring algorithms that can robustly distinguish true biological signals from artifacts. Different computational paradigms—ranging from spectral and statistical models to deep learning frameworks—offer complementary strengths in resolution, robustness, and interpretability, but also exhibit method-specific trade-offs in computational cost and data requirements (Table 3).

#### A/B compartments detection

Detecting A/B compartments from scHi-C data is complicated by sparsity and noise. The Spectral Method with Non-Backtracking Operator was among the first to enable compartment identification at single-cell resolution, using spectral decomposition and maximum correlation entropy to uncover cell-to-cell variability in nuclear organization [90]. Rao *et al*. [10] identified five subcompartments (A1, A2, B1, B2, and B3) in bulk Hi-C; however, the application of such methods to scHi-C is constrained by insufficient interchromosomal contacts and the need for high sequencing depth. To bridge this gap, Xiong *et al*. [30] developed single-cell graph-based Hi-C organization and segmentation toolkit (scGHOST), a graph-based toolkit that utilizes neural networks to segment chromatin contact maps into subcompartments at single-cell resolution. Validated across diverse cell types and 3D genome imaging datasets, scGHOST captures subcompartment dynamics and their associations with transcriptional regulation, offering a scalable solution for subcompartment annotation in single-cell genomics.

#### Topologically associating domains detection

Decoding TADs from ultra-sparse scHi-C data is hindered by low coverage, high stochastic variability, and amplification artifacts. To address this, Li *et al*. [91] proposed deTOKI, a nonnegative matrix factorization-based approach that infers TAD boundaries using low-rank approximations to extract hierarchical interaction patterns from noisy data. While deTOKI demonstrates robust performance in spatial TAD detection, it struggles in contact desert regions.

The existence and stability of hierarchical TAD-like domains (TLDs) at the single-cell level remain an area of active investigation. While some studies report stable hierarchical structures, likely reflecting both biological heterogeneity and method-specific sensitivity. To fill this gap, Li *et al*. [92] developed deDoc2, a dynamic programming-based method for detecting hierarchical TLDs without imputation. This approach revealed that TLDs are dynamic, exhibit cell-cycle-dependent variations, and show heterogeneity across brain cell types. Other methods such as HiCS and scKTLD further enhance boundary resolution and computational efficiency [93, 94]. HiCS detects hierarchical chromatin domains from scHi-C maps and found that numerous transcription factors, chromatin regulators, and histone marks are differentially enriched at domain boundaries, while scKTLD reveals high-frequency TAD-like boundaries associated with conserved population-level domains enriched in architectural proteins like CTCF and Rad21. Moreover, Hua *et al*. [95] introduced DiffDomain, a parametric statistical framework that identifies reorganized TADs by comparing Hi-C contact matrices across biological conditions, offering insights into intra-population chromatin remodeling.

#### Loops detection

SnapHiC first tackled this problem by imputing 10-kb intrachromosomal contact probabilities via RWR, exploiting cell-specific variability to enhance statistical power for chromatin loop identification [96]. SnapHiC2 optimized the same framework, improving processing speed and memory efficiency, and enabling loop identification at 5-kb resolution [97]. This version allows for detailed analysis of promoter–enhancer interactions and prioritization of candidate genes for GWAS variants.

Extending to comparative analysis, Lee *et al*. [98] introduced SnapHiC-D, which identifies differential chromatin contacts across cell types, thus linking 3D chromatin dynamics to functional genomic regulation. Complementing SnapHiC-D, Liu and Ma [99] presented scHiCDiff, a statistical framework that integrates zero-inflated negative binomial regression and nonparametric tests to identify differential chromatin interactions (DCIs) across conditions. This method outperforms existing tools in detecting reproducible DCIs in sparse scHi-C data.

### Feature extraction and dimensionality reduction

After establishing the structural landscape of genomic domains and functional regions, attention shifts to approaches that condense the complexity of high-dimensional scHi-C contact data into compact, informative representations. Feature extraction and dimensionality reduction are pivotal in distilling essential structural patterns from sparse datasets, reducing noise, and enhancing interpretability. Various computational methods have been proposed to learn latent representations that capture cell-type-specific chromatin structures, helping to uncover meaningful biological insights from high-dimensional, sparse contact matrices (Table 3).

#### Topic modeling

To identify cell-type-specific chromatin interaction patterns, latent Dirichlet allocation (LDA)—a classical topic modeling algorithm originally developed for text analysis—has been adapted to scHi-C data [100]. In this framework, each single cell is treated as a “document,” and each chromatin interaction is considered a “word.” This analogy enables the identification of latent topics that represent recurrent chromatin interaction patterns across cells. The scHiC topic modeling utilizes LDA to reveal the relationship between chromatin features and cell-type identity, capturing chromatin domain patterns across cell types and aiding downstream tasks such as clustering and classification [101]. Benchmark analyses suggest that LDA-based approaches perform robustly for datasets with moderate coverage, but require careful tuning of topic numbers to avoid overfitting or loss of resolution.

#### Graph embedding and representation learning

Graph-based representation learning has emerged as a powerful approach for scHi-C analysis, treating contact matrices as adjacency graphs. Bulathsinghalage and Liu [28] employed frequency-based network representations to detect inter-chromosomal hubs, enabling reliable identification of structural motifs despite sparse data. However, this method lacked resolution of hierarchical features like TADs. To address this, scHiCEmbed was developed as an unsupervised graph embedding framework that generates bin-specific latent vectors, preserving structural features such as TAD boundaries and loops [102]. Yet, scHiCEmbed exhibited limited performance under cell-type heterogeneity, leading to the development of scGAD, which incorporates contrastive learning to model inter-cell-type variation, facilitating differential interaction analysis and lineage-specific structural discovery [103]. The latest innovation, scENCORE, integrates graph embedding with neural ODE-based predictive modeling to capture dynamic chromatin conformational changes and provide predictive insights into transcriptional regulation [104].

In comparative evaluations, graph embedding methods generally outperform topic modeling in capturing higher-order and long-range interactions, particularly in heterogeneous cell populations. However, they often require larger training datasets and more computational resources, which may limit their applicability in low-coverage or small-sample studies. Furthermore, existing benchmarks reveal that while scENCORE excels at modeling temporal dynamics, scGAD provides superior resolution in static, multicondition datasets, highlighting the importance of matching the method to the specific analytical goal and data characteristics. In conclusion, these methods highlight the potential of dimensionality reduction techniques to extract biologically relevant features from sparse scHi-C datasets, enabling more effective characterization of chromatin architecture at the single-cell level.

### Cell annotation and clustering

Cell clustering seeks to group cells with similar chromatin architecture, revealing patterns of structural variation across cell types, developmental stages, or conditions, while cell annotation links these groups to known biological categories or functions. Precise cell-type identification based on scHi-C data is fundamental to understanding the principles of chromatin architecture. In response to the sparsity and high dimensionality of scHi-C contact matrics, a range of computational strategies has been introduced to improve classification performance (Table 3).

#### Traditional clustering

Early strategies for scHi-C clustering adapted techniques from bulk Hi-C analysis while addressing single-cell sparsity. As a representative example, scHiCExplorer pioneered a locality-sensitive hashing framework using MinHash to approximate Jaccard similarity between cells [105]. By constructing k-nearest neighbor graphs from compressed interaction fingerprints, this method reduces computational overhead while preserving the integrity of global chromatin architecture features. Subsequent dimensionality reduction via PCA and Uniform Manifold Approximation and Projection (UMAP) enables efficient visualization and clustering of cell populations. However, its reliance on approximate similarity metrics limits resolution for fine-grained cell subtypes, prompting the development of machine learning-driven approaches.

#### Deep learning-based methods

Deep learning approaches have significantly advanced cell-type clustering from scHi-C data. Zhou *et al*. [66] pioneered the scHiCluster algorithm, which applies convolutional smoothing and RWR strategies to denoise sparse contact matrices, followed by PCA and k-means clustering. This demonstrated the feasibility of unsupervised cell-type discovery, although its reliance on linear dimensionality reduction limited its ability to resolve subtle inter-cell-type differences. To overcome this, Wu *et al*. [106] developed scHiCStackL, which incorporates kernel PCA for nonlinear feature extraction, enabling more biologically informative embeddings and improved robustness across heterogeneous populations.

Expanding the scalability of cell-type prediction, SCANN pioneered this domain by leveraging deep neural networks to process sparse chromatin interaction matrices efficiently, demonstrating enhanced training speed and resource efficiency [107]. This prediction method can assist biologists in studying differences in chromosome structure across cell types. While SCANN addressed computational scalability and class imbalance resilience, its reliance on generic feature representations limited biological interpretability for precise annotation. Building on this, CTPredictor introduced multiscale feature engineering to systematically capture hierarchical chromatin patterns [108]. This framework encompasses four innovative feature sets based on the contact matrix, characterized by intuitive interpretability and biological relevance, along with a deep learning model designed to integrate these four feature sets. This feature fusion framework advanced prediction accuracy but remained constrained by its reliance on manually engineered descriptors. Furthermore, scHiClassifier combined four biologically interpretable feature sets with hybrid deep learning architectures that incorporate multihead self-attention and 1D convolutional layer, achieving superior classification performance with enhanced interpretability [109]. By automating the extraction of spatially resolved chromatin features while preserving biological relevance, scHiClassifier outperforms predecessors in classification accuracy and generalizability, as validated through SHAP-based interpretability analyses and functional enrichment studies. Lastly, Fast-Higashi employed tensor decomposition and partial random walks to simultaneously identify cell identity and meta-interactions, linking chromatin structure with cell-type-specific functional states [29]. Unlike traditional deep learning models, Fast-Higashi does not rely on typical neural network architectures; rather, it combines graph-theoretic techniques with tensor-based embeddings to achieve high performance in both clustering and classification tasks. Notably, it bridges structural and functional dimensions of genome architecture by highlighting cell-type-specific chromatin patterns directly from single-cell contact matrices.

This progression from the computational efficiency of SCANN to the feature-aware modeling of CTPredictor, the biologically informed deep learning of scHiClassifier, and the graph-theoretic and tensor-based integration of Fast-Higashi marks a shift in cell annotation. It transforms sparse Hi-C data into interpretable classifiers that reveal the principles of 3D genome organization across diverse cellular states, while bridging structural and functional dimensions by identifying cell-type-specific chromatin patterns directly from single-cell contact matrices.

### Multi-omics integration and functional prediction

Multi-omics integration broadens scHi-C analysis by connecting chromatin architecture with transcriptional activity, epigenetic regulation, and other molecular layers. Its primary aim is to build an integrated framework that clarifies the bidirectional links between 3D genome organization and regulatory processes, thereby enhancing functional interpretation and enabling predictive modeling of cellular phenotypes. This section summarizes computational strategies for combining scHi-C with complementary omics data to gain mechanistic and functional insights (Table 3).

#### Multi-omics integration

The MUDI algorithm represents a significant advancement in integrative analysis by jointly analyzing scHi-C and scRNA-seq data to identify cell subpopulations and topological integration subgroups [110]. By combining these two complementary datasets, MUDI allows for the precise definition of 3D regulatory mechanisms and cell-type-specific interactions, providing a deeper understanding of the biological context in which chromatin interactions occur. This approach not only enhances the identification of cell subtypes but also improves the resolution of chromatin architecture mapping, offering new perspectives on how gene expression is influenced by 3D genome organization.

Another notable integration approach is Muscle, which extends the analysis to include scHi-C, DNA methylation, and chromatin conformation data using semi-nonnegative joint tensor decomposition [111]. This method enables the simultaneous analysis of scHi-C, 3D chromatin conformations, and DNA methylation data at the single-cell level. By utilizing an alternating least-squares algorithm to estimate the parameters of the model, Muscle provides a robust framework for joint analysis of multiple omics layers. The optimality and efficiency of this algorithm make it well suited for large-scale datasets, facilitating the exploration of chromatin regulation and DNA methylation dynamics across different cell types.

#### Functional prediction

Functional prediction based on multi-omics data has seen significant progress with the development of new computational models. The graph transformer-based framework scHiMe pioneers single-cell methylation prediction by integrating DNA sequence data with spatial chromatin interactions derived from scHi-C [112]. Crucially, while sequence inputs alone yield static predictions, incorporating cell-specific Hi-C-derived structural features preserves the cell-type variability, enabling accurate classification of cell types—a performance not achievable with sequence-only models. This finding highlight the unique capacity of scHi-C to capture cell-state-specific methylation patterns, suggesting the bidirectional interplay between 3D genome organization and epigenetic regulation. Building on this framework, scHiGex extends functional prediction to gene expression by modeling chromatin interaction–gene activity relationships through graph transformers [113]. Notably, scHiGex addresses a critical gap in single-cell multi-omics by leveraging spatial constraints of Hi-C data to predict transcriptional outputs, outperforming those from prior bulk-data approaches.

Both tools reveal hierarchical dependencies: scHiMe deciphers how chromatin folding influences locus-specific methylation, while scHiGex elucidates the role of spatial chromatin configurations in orchestrating co-regulated transcriptional programs. By preserving cell-type discriminative power through structure-aware embeddings, these models not only advance beyond mere prediction but also provide mechanistic insights into 3D genome–function relationships and establish chromatin conformation as a linchpin for cell-state annotation [114, 115]. Benchmarking analyses confirm the dual utility of the two frameworks: methylation profiles derived from scHiMe facilitate fine-grained cell clustering, while expression predictions generated by scHiGex reveal lineage-specific regulatory hubs. By integrating their complementary outputs, the two methods reconstitute sparse Hi-C contact maps into the layered and multiresolution framework that can be leveraged to dissect gene-regulatory architecture in detail.

## Analysis and applications of single-cell 3D genomics in mechanistic research

Advances in experimental techniques for single-cell 3D genomics, coupled with increasingly sophisticated computational analyses, have opened new avenues for dissecting the spatial organization of the genome and its role in regulating cellular processes. The central objective in applying these methodologies to mechanistic research is to elucidate the bidirectional relationship between chromatin architecture and transcriptional programs, epigenetic states, as well as environmental cues, under both physiological and pathological conditions. Through the integration of high-resolution chromatin interaction data with complementary molecular layers, it becomes possible to uncover structural signatures linked to cell identity, developmental transitions, and disease-specific alterations.

**Figure 3:**
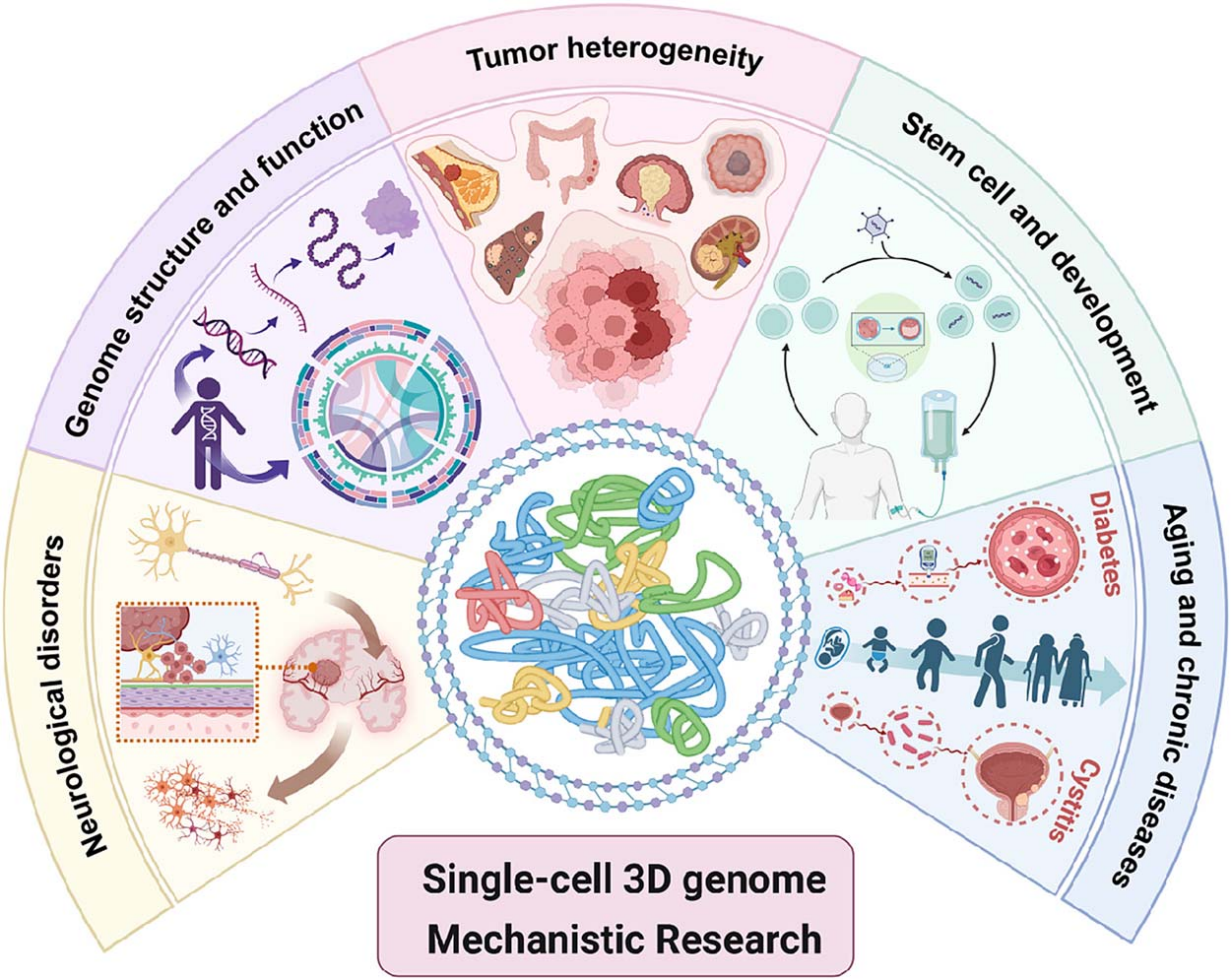
Framework for single-cell 3D genome mechanistic research, spanning biological domains such as genome structure, cancer heterogeneity, ESCs, neurological disorders, aging, and chronic diseases.

This section examines the application of single-cell 3D genomic approaches across diverse biological systems, including cancer, stem cell differentiation, immune regulation, and neurological development (Fig. 3). By aligning experimental platforms with tailored computational workflows, these studies reveal how technological choices influence the resolution, interpretability, and reproducibility of biological insights (Table 4). The discussion emphasizes a cohesive connection between the generation of single-cell 3D genome data and its translation into mechanistic understanding, thereby providing a framework for leveraging these innovations to address complex questions in functional genomics.

**Table 4:** Applications of single-cell 3D genomics in mechanistic research, categorizing key biological and disease-related contexts.

Biomechanism	Primary goals	Technologies and tools used	Main findings	Limitations
Genome structure and function	Understand chromatin architecture dynamics, epigenetic–structural interactions, and developmental regulation across species	scHi-C, scMicro-C, SnapHiC2, scHiCDiff, Higashi, scHiCAtt, CRISPR-based perturbation mapping, single-cell multi-omics integration	Identified stochastic TAD establishment; resolved stable versus transient loops; mapped allele-specific methylation-dependent loops; linked chromatin plasticity to transcriptional noise; validated loop anchors for pluripotency	Low-depth data risk missing transient loops; computational sensitivity critical for rare events
Cancer heterogeneity	Reveal how chromatin topology drives tumor evolution, heterogeneity, and therapy resistance	scHi-C, multi-omics integration, structural instability index, CTCF/cohesin depletion models	Discovered subtype-specific enhancer hijacking; mapped hypoxia-responsive domains; identified fused chromatin domains linked to invasion; preserved stem-like topology in AML relapse clones	Need for higher resolution in solid tumor samples; clinical translation limited by data sparsity
Neurological disorders	Uncover chromatin structural disruptions underlying neurodegeneration and psychiatric disease	Droplet Hi-C, Pop-C, vDip-C, scGHOST, single-cell transcriptomics integration	Detected ecDNA in AD neurons; mapped cerebellar aging-related reorganizations; identified prefrontal cortex chromatin hubs linked to autism and schizophrenia	High sequencing depth requirements; noise filtering essential; limited coverage of diverse brain regions/stages
Aging and chronic diseases	Link 3D genome instability to aging-related degeneration and chronic pathologies	scHi-C, Micro-C, deDoc2, single-cell multi-omics	Identified TAD loss and compartment shifts in aging; linked SIRT1 decline to inflammation in cystitis; connected β-cell chromatin reorganization to diabetes	Need for rare cell detection; integration of structural and functional data in clinical samples remains challenging
ESCs and development	Explore 3D genome regulation of pluripotency, reprogramming, and regeneration	scMicro-C, Dip-C, allele-resolved Hi-C, interspecies haESC models	Mapped TAD formation preceding pluripotency gene activation; showed species-specific loops affecting metabolism; linked mitochondrial dysfunction to lamina–chromatin disruption and prosenescence gene activation	Cross-species normalization needed; require integration with proteomics and real-time imaging

### Advancements in uncovering genomic structure and function

Single-cell 3D genomics has revolutionized our understanding of genome architecture by revealing chromatin organization and regulatory roles. An early scHi-C study in *Drosophila melanogaster* revealed that TAD formation coincides with zygotic genome activation, suggesting that 3D chromatin structure orchestrates lineage-specific transcriptional programs [116]. Notably, cell-to-cell variability in TAD establishment indicates that stochastic chromatin folding may underlie divergent developmental trajectories.

The power of single-cell 3D genomics is further exemplified by its ability to resolve fine-scale chromatin interactions. When integrated with computational loop-calling algorithms such as SnapHiC2 or scHiCDiff, these high-resolution datasets enable precise discrimination between stable structural anchors and transient contacts, which is crucial in systems with rapid cell state transitions such as stem cell differentiation [97, 98]. Cross-species comparisons further demonstrate the evolutionary conservation of folding principles, highlighting shared strategies for developmental gene activation despite genomic divergence—underscoring the universality of 3D genome regulation in embryogenesis and differentiation [20, 81, 116, 117].

Single-cell approaches have also elucidated epigenetic–structural interactions. For example, allele-specific DNA methylation governs parent-of-origin chromatin loops, ensuring monoallelic expression. In disease contexts, hypomethylation-induced loop destabilization facilitates aberrant oncogene activation in rare human premalignant hematopoietic cells [24]. The integration of single-cell multi-omics further illuminates how chromatin spatial organization adapts to functional demands [118]. By simultaneously mapping chromatin conformation, histone modifications, and transcriptional output within individual cells, studies have shown that A/B compartmentalization varies dynamically across cells, linking chromatin spatial heterogeneity to lineage-specific differentiation trajectories.

Chromatin plasticity within clonal populations has also been redefined by single-cell 3D profiling, demonstrating how structural heterogeneity underpins stochastic gene expression [60]. Here, computational models such as Higashi and scHiCAtt outperform traditional imputation methods in predicting long-range enhancer–promoter variability that drives transcriptional noise [29, 73]. Machine learning models have predicted transcriptional bursting events directly from chromatin spatial features at the single-cell level [119]. Moreover, CRISPR-based screens guided by single-cell 3D maps have validated loop anchor regions essential for maintaining pluripotency, emphasizing causal structure–function relationships at cellular resolution [120]. This experimental–computational synergy highlights how targeted perturbations informed by structural maps can be used to test causality, moving beyond correlative observations.

In developmental systems, such as mouse embryogenesis, single-cell analyses have captured dynamic chromatin reorganizations across blastomeres, priming lineage-specific transcription programs [43]. In mouse pluripotent stem cells, single-cell multi-omics integration has uncovered chromatin loop dynamics essential for maintaining pluripotency, often missed in bulk analyses [81]. Furthermore, rare cell subpopulations exhibiting collapsed TAD boundaries and premature activation of differentiation genes have been identified, illustrating how structural heterogeneity buffers against premature fate determination. Importantly, these developmental insights depend on both experimental resolution and computational sensitivity—low-depth data risk missing transient but functionally critical loops.

Collectively, these advances position single-cell 3D genomics as a transformative tool for decoding the structural basis of gene regulation. By correlating structural variability with functional outcomes at cellular resolution, this paradigm shift illuminates previously unrecognized regulatory checkpoints (Fig. 3 and Table 4).

### Decoding cancer heterogeneity

Single-cell 3D genomics has emerged as a pivotal tool for dissecting the spatial and temporal complexity of cancer evolution, revealing how chromatin architecture fuels tumor heterogeneity across diverse malignancies. In renal cell carcinoma, single-cell chromatin conformation maps uncovered subtype-specific enhancer hijacking events, where dysregulated chromatin loops aberrantly connect oncogenic enhancers to promoters, driving transcriptional programs unique to aggressive tumor subclones. Similarly, hepatocellular carcinoma exhibits spatially coordinated chromatin domains that mediate hypoxia-responsive gene activation, with single-cell resolution exposing how intratumoral structural variability correlates with metabolic plasticity and drug resistance. These findings underscore the role of 3D genome dysregulation in shaping phenotypic diversity within solid tumors [121, 122].

Breast cancer studies further illustrate the functional consequences of chromatin rewiring [123, 124]. Single-cell multi-omics analyses revealed that hormone receptor-positive and triple-negative subtypes develop distinct 3D interactomes, with estrogen receptor-bound chromatin loops dynamically collapsing during endocrine therapy resistance. In pancreatic ductal adenocarcinoma, scHi-C identified metastatic subpopulations harboring fused chromatin domains that coordinate pro-invasion gene modules, demonstrating how structural alterations precede overt transcriptional changes [125]. Glioblastoma and colorectal cancers add another layer of complexity, where vascular mimicry-associated tumor cells exhibit unique chromatin compartmentalization patterns that activate transcriptional programs undetectable in bulk assays [53]. These findings suggest that the evolutionary trajectories of hematological malignancies are likewise governed by 3D genome dynamics. Besides, in hematologic malignancies such as acute myeloid leukemia (AML), relapse-driving subclones preserve stem-like 3D topologies resistant to therapy-induced disruption [126]. These findings collectively highlight 3D genome architecture as a unifying principle underpinning intratumoral heterogeneity, therapeutic resistance, and malignant evolution across cancer types.

These insights underscore the therapeutic potential of restoring chromatin topology or targeting oncogenic hubs to combat tumor adaptability (Fig. 3 and Table 4). Single-cell 3D genomics thus provides a structural framework for precision oncology, illuminating the architectural basis of cancer progression and resilience, but further advancement will require the development and integration of more sophisticated experimental and computational platforms to support continued research.

### Unraveling neurological disorders: brain pathogenesis

Recent breakthroughs in single-cell 3D genomics have provided critical insights into the pathogenesis of neurological disorders. By capturing the spatial conformation and dynamic remodeling of chromatin at single-cell resolution, these approaches elucidate the complex relationship between genome structure and gene expression regulation, shedding light on neurodevelopment, aging, and degenerative diseases.

In neurodegenerative diseases such as Alzheimer’s disease (AD), single-cell 3D genomics has uncovered chromatin structural abnormalities linked to neuronal dysfunction. For instance, Wang *et al*. [127] developed Droplet Hi-C for high-throughput single-cell chromatin conformation profiling, revealing the presence of ecDNA, which is associated with genomic instability and drug resistance in AD neurons. Compared with traditional scHi-C, droplet-based methods provide better scalability and capture rare neuron subtypes, but require substantial sequencing depth and advanced noise-filtering algorithms to avoid false positives in ecDNA detection. Regarding brain region-specific pathological mechanisms, the team at Stanford developed population-scale Dip-C (Pop-C) and virus-enriched Dip-C (vDip-C) technologies, which mapped the 3D genome of the cerebellum, revealing age-related reorganizations. These findings suggest that 3D genome stability may protect against neurodegeneration [128]. Furthermore, the scGHOST algorithm identified cell type-specific chromatin interaction hubs in the prefrontal cortex, linking 3D genome disruptions to epigenetic abnormalities in psychiatric disorders like autism [30]. Integrating this method with single-cell transcriptomic data also establishes associations between chromatin spatial positioning and gene expression dynamics, providing new tools for elucidating molecular networks in schizophrenia and other diseases.

In summary, single-cell 3D genomics is transforming the understanding of neurological disease mechanisms, especially when experimental resolution is matched with computational methods optimized for sparse and heterogeneous neural datasets. It offers new diagnostic biomarkers and therapeutic targets, paving the way for precision medicine in neurodegenerative diseases and mental disorders (Fig. 3 and Table 4). Future progress will require not only broader inclusion of diverse brain regions and disease stages, but also the development of more advanced experimental and computational platforms to capture transient chromatin states that may underlie early disease onset.

### Aging and chronic diseases

Aging and chronic diseases are interconnected through the progressive accumulation of molecular damage, with the destabilization of the 3D genome architecture being central to this relationship [129–131]. As organisms age, chromatin integrity declines due to oxidative stress, epigenetic drift, and reduced DNA repair, predisposing tissues to chronic diseases by disrupting gene regulatory networks. For example, age-associated 3D genome disorganization not only accelerates cellular senescence but also primes metabolic tissues like pancreatic islets for dysfunction, fostering diabetes progression in the adult mouse brain, and aged human pancreatic islets [54, 130]. Similarly, chronic inflammatory conditions such as cystitis also exhibit overlapping molecular features with aging, including aberrant chromatin folding patterns that sustain pathological gene expression loops in human bladder tissue from patients with interstitial cystitis [132]. These shared mechanisms further link the 3D genome to aging and chronic disease pathogenesis.

The 3D architecture of the genome plays a pivotal role in maintaining cellular homeostasis, and its disruption has emerged as a critical factor in aging and chronic diseases. Single-cell 3D genomics, which enables the resolution of chromatin interactions at cellular resolution, has revolutionized the understanding of how genomic spatial organization influences age-related degeneration and chronic pathologies [54, 129]. During aging, the 3D genome undergoes progressive disorganization, characterized by the loss of TADs and altered chromatin compartmentalization [133]. These structural changes correlate with transcriptional dysregulation involved in cellular senescence, DNA repair, and metabolic pathways. These observations were made possible by single-cell 3D genomics combined with computational domain-calling algorithms (e.g. deDoc2) that can detect subtle TAD boundary shifts in inflamed versus aged tissues [92]. In addition, single-cell 3D genomics shows that cystitis involves chromatin reorganization of inflammatory genes, disrupting regulatory connectivity, and driving chronic inflammation, with age-related declines in chromatin remodelers like SIRT1 exacerbating the process [59, 132]. Similarly, perturbations in chromatin folding in pancreatic β-cells have been linked to impaired insulin secretion in diabetes, highlighting the role of 3D genome instability in metabolic disorders [130].

These advancements underscore the importance of preserving 3D genome integrity as a strategy to mitigate aging and chronic disease progression (Fig. 3 and Table 4). Going forward, translating these insights into clinical interventions will require the development of more advanced experimental platforms capable of resolving transient chromatin states in rare cell populations, as well as computational frameworks that can integrate structural, transcriptomic, and epigenetic data from heterogeneous clinical samples.

### Embryonic stem cells and development

Single-cell 3D genomics has become essential for understanding the mechanisms behind embryonic stem cell (ESC) development and cell fate decisions [134, 135]. By providing high-resolution chromatin architecture mapping, these technologies reveal how spatial genome organization influences ESC pluripotency and differentiation. In particular, scMicro-C and Dip-C, applied to both *Mus musculus* and human ESCs, have enabled the detection of fine-scale enhancer–promoter loops critical for maintaining pluripotency, which are often undetectable in bulk Hi-C datasets [52, 57]. In the context of ESC development, studies have shown that in mouse embryos, *de novo* TAD formation precedes the activation of pluripotency genes, such as Oct4 and Nanog [43, 136]. Moreover, somatic cell reprogramming involves dissolving somatic-specific chromatin loops to establish ESC-like interaction hubs [137, 138]. Epigenetic modifications, such as DNA methylation suppression, accelerate TADs reconfiguration, linking epigenetic changes to 3D genome plasticity.

In addition, the generation of haploid ESCs (haESCs) provides a unique model to study 3D genome symmetry-breaking. Research by Sun *et al*. showed that mouse–rat inter-species allodiploid ESCs retain species-specific chromatin compartmentalization despite nuclear fusion [116]. Using allele-resolved Hi-C, they identified conserved enhancer–promoter loops regulating pluripotency (e.g. Sox2), while species-specific loops governed divergent metabolic pathways. This work underscores how interspecies chromatin architecture divergence influences evolutionary adaptability and reprogramming efficiency. A groundbreaking study uncovered that mitochondrial dysfunction in aged stem cells induces aberrant DNA methylation at lamina-associated domains, disrupting nuclear lamina–chromatin interactions [139]. This led to ectopic activation of prosenescence genes and impaired tissue regeneration in murine models. These findings position metabolic–epigenetic crosstalk as a therapeutic target for age-related regeneration deficits, demonstrating how single-cell 3D genome dynamics are not only critical for ESC development but also pivotal in tissue regeneration and development. These findings demonstrate how single-cell 3D genome dynamics are not only critical for ESC development but also pivotal in tissue regeneration and organismal development.

Overall, recent advancements in single-cell 3D genomics have provided profound insights into the mechanisms underlying ESC development, cell fate decisions, and the dynamic reorganization of chromatin (Fig. 3 and Table 4). These findings show that single-cell 3D genomics is essential for ESC biology and regenerative medicine. Future progress in regenerative medicine will require more advanced experimental and computational platforms capable of integrating genome topology with transcriptomic, epigenomic, and proteomic data to fully understand and manipulate developmental programs.

## Discussion

The emergence of single-cell 3D genomics has fundamentally reshaped the understanding of chromatin architecture, offering a dynamic lens through which to explore the spatial and temporal organization of the genome at unprecedented resolution. By integrating cutting-edge experimental techniques with advanced computational methodologies, this field has unraveled the intricate interplay between chromatin folding [48], gene regulation [3], and cellular heterogeneity [140].

Experimental innovations such as scHi-C and its derivatives have enabled genome-wide chromatin interaction profiling at single-cell resolution, overcoming the limitations of bulk-cell approaches that obscure cell-to-cell variability. These advancements are complemented by multi-omics platforms like HiRES [43] and LiMCA [141], which reveal how spatial genome organization directly influences gene expression programs. Computational tools have further enhanced the ability to interpret sparse and noisy single-cell data, enabling robust reconstructions of 3D chromatin conformations and predictive modeling of regulatory dynamics. Studies in cancer [53, 121], neurobiology [127], and developmental [35, 116] highlight chromatin misfolding in oncogene activation, spatial genome disorganization in neurodegenerative, and chromatin reconfiguration in aging—positioning the 3D genome as a key regulator of cellular identity and function.

Despite progress, challenges remain in single-cell 3D genomics, including low throughput, high sequencing costs, and limited interpretability of computational models. These limitations hinder large-scale studies, particularly in rare cell populations or clinical samples, and restrict broader application in understudied systems. Future advancements should focus on multimodal integration, combining chromatin conformation with proteomics and transcriptomics, and real-time chromatin tracking. These innovations will enhance understanding of dynamic processes, moving 3D genomics toward predictive tools for precision medicine.

## Conclusion

Single-cell 3D genomics has become central to modern biology, redefining the understanding of genome organization and its functional implications. By disentangling the interplay between chromatin architecture, epigenetic states, and transcriptional outputs, this field has illuminated the molecular logic of cellular heterogeneity, disease progression, and developmental programming. As experimental and computational tools continue to mature, this field is poised to revolutionize both basic science and translational medicine.

**Key Points**

- Experimental innovations in single-cell 3D genomics have enabled high-resolution, genome-wide profiling of chromatin architecture at single-cell level.
- Advanced computational frameworks support all stages of single-cell 3D genomics analysis—including data preprocessing, structural reconstruction, and functional annotation—thus enabling robust interpretation of complex chromatin landscapes.
- Applications of single-cell 3D genomics have uncovered dynamic chromatin mechanisms in genome biology, development, and disease, revealing the structural basis of gene regulation, cellular heterogeneity, and pathological transitions.
